# Anchorage Research for CFRP Tendons: A Review

**DOI:** 10.3390/ma17133208

**Published:** 2024-07-01

**Authors:** Yalong Li, Taining Shi, Yafeng Qiu, Yuanlin Zhu, Longkang Zhang

**Affiliations:** 1 Interdisciplinary Center for Fundamental and Frontier Sciences, Nanjing University of Science and Technology, Nanjing 210094, China; 2 Fasten Hopesun Group Co., Ltd., Jiangyin 214433, China

**Keywords:** anchors for CFRP tendons, damage mechanism, testing techniques, anchorage performance under non-ideal conditions

## Abstract

Carbon fiber reinforced polymer (CFRP) tendons are composite materials that offer significant advantages in terms of tensile strength and lightweight properties. They are being increasingly utilized in the construction industry, particularly in bridge cables and building structures. However, due to their relatively poor transverse mechanical properties compared to steel cables, securing these tendons with anchors presents a challenge. This paper reviews the structure and force characteristics of three types of anchors for CFRP tendons—clamping anchorage, bonded anchorage, and composite anchorage—analyzes and summarizes the anchorage characteristics and damage mechanisms of each type of anchorage, and highlights that the optimization of the mechanical properties of the tendons is key to the design and research of anchoring systems. The new composite anchorage offers comprehensive advantages, such as minimal tendon damage at the anchorage section, more uniform stress distribution, and better anchorage performance, despite being more complex in design compared to single-type anchorages. However, there remain challenges and research gaps in testing and validating these anchoring systems under realistic loading and environmental conditions, including impacts, cyclic stresses, humidity, and high temperatures. Future efforts should focus on developing new testing techniques and models to simulate real-world conditions, enabling more accurate assessments of anchorage performance and longevity. By doing so, we can fully harness the mechanical properties of CFRP tendons and further enhance the safety and efficiency of our built environment.

## 1. Introduction

Carbon fiber reinforced polymer (CFRP) is an emerging non-metallic composite material recognized for its impressive hardness, resilience, light weight, and resistance to corrosion. Traditionally, CFRP is produced through the high-temperature oxidation and carbonization of organic fibers, which are then interwoven with an epoxy resin matrix. This innovative reinforcing fiber leads to the creation of CFRP tendons. With a density approximately one-fifth of that of steel, their tensile strength can reach up to ten times that of steel, aligning with the concept of lightweighting in modern construction [1]. They have been hailed as an ideal replacement for conventional steel cables in bridge engineering. The superior mechanical properties of these tendons offer promising opportunities for constructing large-span bridges [2,3]. Beyond bridge construction, CFRP tendons have been employed in structural reinforcement to augment load-carrying capacity in building construction and the remediation of high-risk buildings [4,5,6].

Tensile tests on CFRP bars reveal a fracture damage pattern progressing from the exterior to the interior of the bar. Unlike traditional steel bars [7], they display a nearly linear stress–strain curve without a yielding stage, indicating brittle damage. However, CFRP exhibits anisotropy, leading to relatively poor radial mechanical properties in CFRP tendons. Their radial compressive strength is about 160 MPa, significantly lower than their longitudinal tensile strength, posing a substantial challenge for anchoring CFRP tendons. The performance of CFRP tendon anchorages can significantly impact the overall mechanical properties of the tendons; as Nanni [8] demonstrated, the ultimate load-bearing capacity of a structure equipped with CFRP prestressing tendons depends more on the anchorage system’s performance than on the tendon strength.

Therefore, investing in anchorage mechanisms and developing new types of anchorages are crucial for the engineering applications of CFRP tendons. This paper provides a comprehensive description of the anchoring systems used with various types of CFRP bars and delivers an in-depth analysis of the damage mechanisms associated with each type of anchorage. Additionally, we present the challenges encountered during the development, testing, and validation of new CFRP reinforcement anchorage devices by exploring novel detection techniques for measuring anchorage stress, strain, and damage, as well as the performance of these anchorages under less-than-ideal conditions. It is hoped that this work will guide the design of innovative anchorage devices and enhance overall anchorage performance.

## 2. Research Status of CFRP Reinforcement Cable Anchorage

In the context of bridge cable anchorage, medium carbon alloy steel is typically employed in order to ensure the comprehensive mechanical properties and processing performance that are required. Commonly used medium carbon alloy steel includes 40 Cr, 35 CrMo, 42 CrMo, and 40 CrNiMo. Table 1 illustrates the characteristics and applicable occasions of these steels.

Traditional CFRP tendons anchorage is divided into three main categories: mechanical clamping anchorage, sleeve bonded anchorage, and composite anchorage. Their anchorage mechanism and anchorage damage mechanism have their own characteristics.

### 2.1. Mechanical Clamping Anchorage

**Figure 1:**
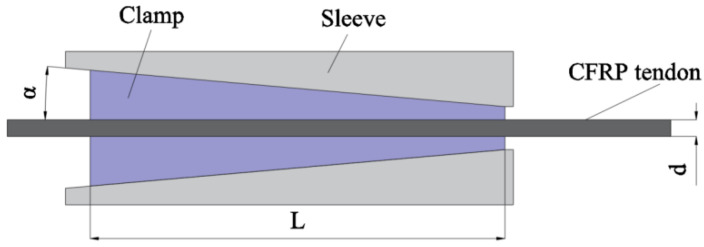
Mechanical clamping anchorage.

Mechanical clamping anchorage, as depicted in Figure 1, is a type of anchorage system that boasts a simpler design and is commonly used for securing regular tendons. Its construction process is straightforward, reusable, and cost-effective, while typically comprising an external anchor sleeve and a clamping piece. The strength of the clamp is less than that of the sleeve. It is typically manufactured from 45 steel, which is an elasto-plastic reinforced material.

The anchoring mechanism operates as follows: the internal pressure applied by the clamp onto the surface of the carbon fiber reinforced polymer (CFRP) tendons induces a gripping action. Concurrently, the friction at the clamp–tendon interface inhibits tendon slippage. This mechanism is generally adopted for single tendon anchoring. In this form of anchorage, the tendons undergo significant extrusion and shear stresses generated by friction [9]. To prevent slippage during use, substantial radial compressive stress must be applied—a strategy that may lead to potential damages in the form of extrusion and excessive biting due to CFRP tendons’ poor transversal mechanical properties.

To mitigate excessive biting, tendons are often encased in aluminum or copper tubes [10] and positioned within wedge-shaped clamps. Both types of tubes, possessing low yield strength and hardness, form-fit the tendon’s surface through yield deformation during pulling and drawing, thereby reducing tendon biting. This method is relatively straightforward to implement, yet it offers less comprehensive protection for the tendon material than bonded anchorage. Some studies suggest sandblasting the tendon’s surface in the anchorage region before placing it into the fixture wedge. This increases the friction at the force interface by upping the friction coefficient and reducing slippage while decreasing the magnitude of compressive stress, ultimately improving the anchorage effect. However, it does not alter the complex stress distribution state within the force interface. Moreover, stress in the anchorage area generally concentrates towards the loading end. Al-Mayah et al. [11] suggested the concept of a variable inclination angle sleeve clamping type of anchorage to alleviate this stress concentration. This is achieved by varying the inner surface’s inclination at different positions, transforming it from a straight to a circular arc surface. However, it increases the cost of processing.

Zhuge et al. [12] introduced a novel mechanical anchorage design that takes into account the compressive stress distribution and contact surface friction of the clamping anchorage. To minimize uneven compressive stress distribution within the anchorage area, the clamp’s cone angle was made slightly larger than the sleeve’s cone angle. The angular difference causes a gap between the anchor sleeve and the clamp at the loading end, which prevents excessive compressive stresses at the loading end. However, it is necessary to maintain a certain degree of alignment between the clamp and the anchor sleeve during assembly, otherwise the CFRP tendon will experience tensile bending stress when subjected to force.

In conclusion, while treating the surface of tendons and optimizing the structural parameters of the clamping anchorage may improve the stress state in the anchorage zone to some extent, this method demands high assembly precision. Misalignment of tendons may accelerate anchorage failure. Despite these adjustments, the loaded tendons must still withstand significant clamp pressure, posing a risk of compression collapse and resulting in poor fatigue performance. Therefore, this approach is not suited for usage under high-load conditions, nor does it fully realize the excellent tensile performance of tendons. Furthermore, this technique is generally only applicable for single tendon anchoring—it proves challenging to ensure even force distribution across multiple tendons in a cable comprising dozens or even hundreds of CFRP tendons.

### 2.2. Bonded Anchorage

#### 2.2.1. Research on Conventional Bonded Anchorage

Bonded anchorage, comprising a bonding medium and sleeve, is the most extensively researched and applied form of anchorage. The composition of the bonding medium is not fixed. It is typically composed of epoxy resin as a base, mixed with mortar, iron sand, and hardener. Epoxy resin is a polymer. Due to the chemical activity of the epoxy group, a variety of compounds containing active hydrogen can be used to implement ring-opening, curing, and cross-linking to generate a network structure, with good adhesive strength. The anchoring mechanism of the bonded anchorage primarily relies on the chemical adhesion between the bonding medium and CFRP tendons, supplemented by friction and the biting force between the bonding medium and the wall of the sleeve to transfer shear force. This arrangement effectively prevents tendon damage. The inner cavity design of the traditional bonded anchorage sleeve has evolved from a straight to an inner cone and then to a straight cone type.

**Figure 2:**
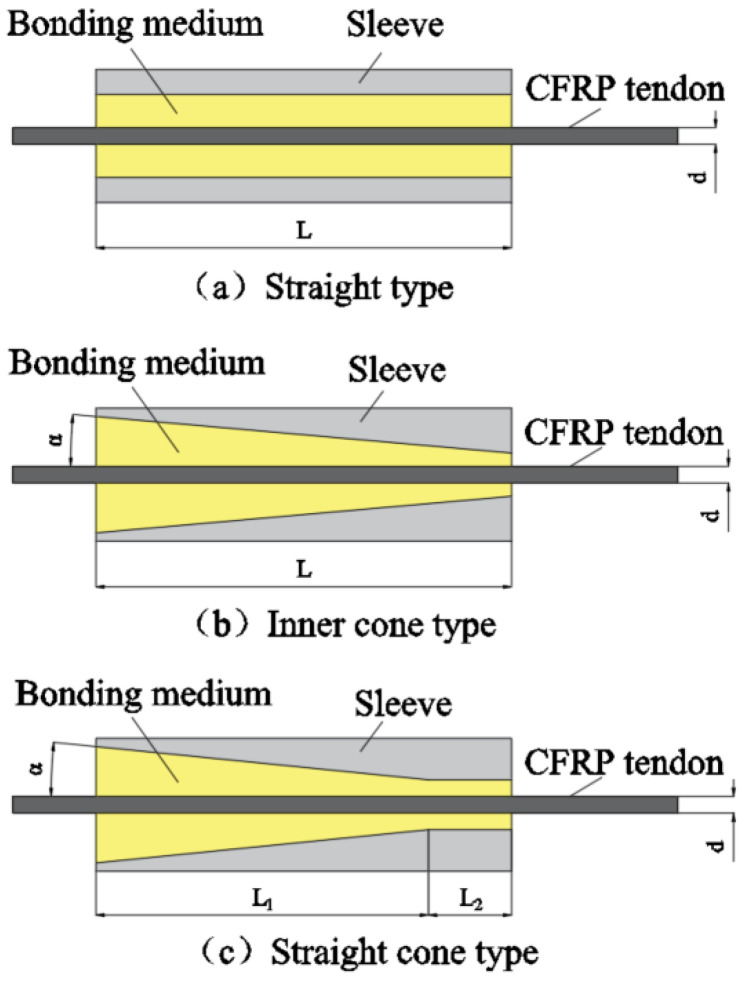
Conventional bonded anchorage.

In the straight anchorage illustrated in Figure 2a, the tensile force exerted on the CFRP tendons is primarily sustained by the chemical adhesion at the tendon–adhesive interface. Zhang et al. [13] conducted an experimental study on the anchoring of CFRP tendons in straight anchorage and discovered that the bond stress at the tendon–adhesive interface is not uniformly distributed across the adhesive length. The peak point of bond strength is proximate to the loading end of the anchorage under minimal load, moving towards the free end as the load on the tendons increases. A small residual bond stress remains at the loading end. Furthermore, the distribution of bond stress relates to the anchorage length: shorter lengths yield a more uniform bond stress distribution, while longer lengths result in uneven stress distribution. The straight type anchorage has large length dimensions, which are not favorable for transportation and installation.

To enhance the residual bond strength at the loading end, an inner cone anchorage design was proposed as depicted in Figure 2b. Here, the tendons transfer the applied tension force in the inner cone anchorage to the bonding medium via adhesion at the tendon–adhesive interface. This force is then applied to the inner surface of the conical anchor sleeve, generating a radial squeezing force which enhances the friction and hence residual bond strength at the medium-anchor sleeve interface. Increasing the cone’s inclination angle and the anchorage length improves the anchorage’s ultimate load capacity. A moderately large inclination angle boosts the bond strength at the tendon–adhesive interface and decreases the critical anchorage length. An excessive angle can lead to shear failure at the loading end. Generally, an angle of 3° is ideal [14].

In response to the issue of stress concentration at the inner cone anchorage loading end, Mei et al. [15] proposed a straight cone anchorage design. As shown in Figure 2c, this anchorage’s inner cavity is the combination of a cone and a straight cylinder. The straight cylinder section provides a transition, with peak extrusion stress occurring at the bend corner of the straight cone and decreasing towards the loading end. Tensile tests conducted on these anchors showed that all tendon damage modes were filamentary pull-off damages, suggesting high anchorage efficiency [16]. Based on this, Liu [17] also added a small straight section at the free end and analyzed the contact stress and friction between the scheme and the inner wall of the straight cone anchorage sleeve by ANSYS simulation, and concluded that the addition of a straight section at the end of the anchorage is conducive to increasing the compressive stress in the inner wall of the anchorage section and transferring the friction of the bonding colloid to the loading end. The straight cone type of anchorage shows compressive stress concentration at the straight cone connection, which reduces the tendon damage at the loaded end to some extent, but the compressive stress in the anchored section is still not uniformly distributed.

#### 2.2.2. Research on New Type of Bonded Anchorage

**Figure 3:**
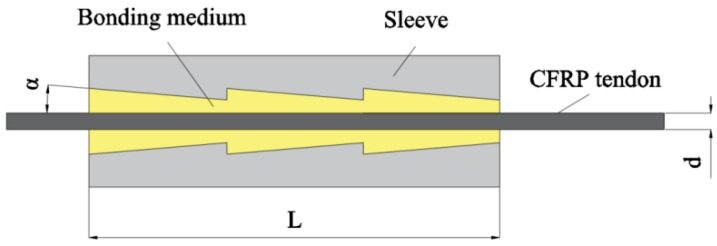
Multistage cone anchorage.

Conventional cone-bonded anchorage is characterized by a large radial size, with its outer diameter being influenced by the diameter of the inner cavity opening at the free end. This opening gradually enlarges from the loading end to the free end. When anchoring large-diameter tendons or multiple tendon bundles, longer anchorage lengths are often necessary. However, an increase in anchorage length further augments the radial size of the cone-bonded anchorage, leading to a larger overall size. This is not advantageous for applying and promoting CFRP tendons. To address this issue, Zhu et al. [18] suggested designing a multi-stage cone anchorage, as shown in Figure 3.

The internal cavity of this anchorage connects several cones. The radius of the large end of each cone segment remains consistent, and the radial size of the anchorage is regulated accordingly. This design significantly reduces the size. The multi-stage cone structure reduces the variation of the thickness of the bonding medium in the sleeve, which balances the radial strain of the anchoring section to a certain extent. Therefore, its compressive stress distribution is more uniform than that of traditional bonded anchorages. Wang et al. [19] conducted experimental research on the anchorage mechanism and fracture forms of CFRP tensile cables anchored by multi-stage cone anchorage. They proposed a finite element simulation method based on the bond-slip behavior of the tendon–adhesive interface, which aligned with actual scenarios. Finally, optimization analysis revealed that moderately increasing the anchorage length could significantly enhance its efficiency.

**Figure 4:**
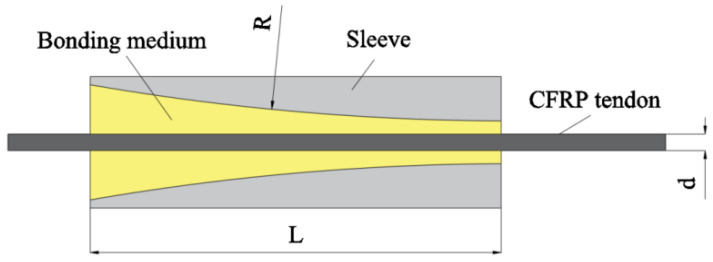
Bow anchorage.

In studying the stress distribution of inner cone anchorage, Ju et al. [20] designed and proposed a bow anchorage to alleviate stress concentration at the loading end, based on the inner cone anchorage. The structure of the anchorage is shown in Figure 4.

An experimental comparison between the bow anchorage and the cone anchorage [21] revealed that its ultimate load capacity generally exceeds that of the traditional cone anchorage. Furthermore, it effectively mitigates stress concentration and reduces peak stress at the loading end. The ultimate load capacity of this anchorage is related to the slip between the medium and the anchor sleeve. Within a certain range, the smaller the inclination angle of the anchor sleeve, the larger is the slip. Jia et al. [22] designed an anchorage with an inner cavity shape of cone + arc + straight, which reduces stress concentration by converting sharp corners at the transition into arcs based on the cone-straight anchorage. Nevertheless, the radial dimensions of curved structures exhibit greater variability and are typically larger than those of tapered anchors for a given length of anchorage.

**Figure 5:**
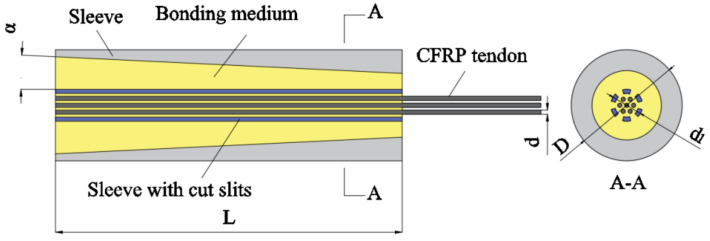
Double-cylinder bonded anchorage.

To overcome issues such as the uneven distribution of extrusion stresses in the internal wire strands of the inner cone anchorage and the existence of bending at the loading end, Hou et al. [23] suggested a double-sleeve bonded type of anchorage, as depicted in Figure 5. Compared with the conventional inner cone anchorage, this type has a steel sleeve with multiple longitudinal cut slits on the tendon surface. The width of these slits varies gradually along the longitudinal direction, meaning that the casing stiffness also gradually changes during radial contraction. Experimental findings indicate that this anchorage avoids excessive extrusion stress at the loading end, offers reasonable stress distribution, and achieves approximately 30% higher anchoring efficiency than inner cone anchorage. Nevertheless, the inner casing is more challenging to machine, and the anchorage is manufactured with additional assurance of its coaxiality.

#### 2.2.3. Other Bonded Anchorage

In addition to studying new anchors with structural modifications, researchers have also explored new anchors by changing the parameters of the components of bonded anchors. Typically, these types of anchorages are based on traditional bonded anchorages with alterations made to the stiffness of the bonding medium, or they improve the surface contact effect of tendons or the state of tendons in the bonding medium to achieve superior anchorage performance.

Meier et al. [24] used a computer-aided material design combined with alumina ceramics and epoxy resin to develop a variable stiffness bonding material. The anchor cylinder structure is internally cone-shaped, with the modulus of elasticity of the bonding material being low at the loading end and increasing as it nears the free end. The elasticity modulus was altered by coating alumina ceramic particles with various thicknesses of epoxy resin, with thicker coatings resulting in a lower modulus. Experimental validation demonstrated that this material effectively reduces peak stress at the loading end, achieves more uniform stress distribution, and provides excellent anchoring effects.

**Figure 6:**
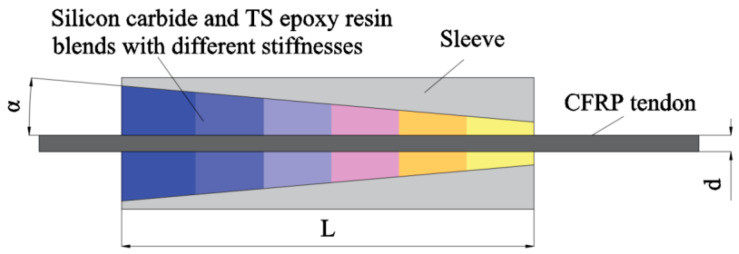
Variable stiffness bonded anchorage.

Niu et al. [25] prepared bonding materials with varying stiffness by blending silicon carbide powder and TS epoxy resin in different mass ratios. After curing, these were applied to the bonded anchorage, as illustrated in Figure 6. Simulation demonstrated that as the number of stiffness values increased, stress distribution in the anchorage area became more uniform, effectively preventing local tendon fractures. However, creating the bonding material requires a certain curing time, and the higher the number of stiffness values, the longer is the preparation time. Thus, the optimal number of stiffness values was experimentally analyzed and determined to be six.

Increasing the bond strength between tendons and the bonding medium can effectively transfer the shear force. The bonding stress at the interface comprises two components: chemical adhesion and friction. Chemical adhesion depends on the material properties of the bonding medium and tendons. Due to the presence of epoxy resin in both the bonding medium and the CFRP tendon, the two are more compatible and have a stronger chemical bond. Friction depends on the surface roughness and radial compression force. Increasing friction through enhancing surface roughness is a simple and feasible way to boost bond stress. Jung et al. [26] found experimentally that sand coating or oxide coating could triple bond strength and significantly shorten the anchorage length. Hassoon et al. [27] proposed two new methods to elevate bond stress on the surface of CFRP tendons by using epoxy resin as a binder and coating the tendon surface with sand and steel fibers, thereby improving the surface contact effect of the tendons. These two reinforcement techniques enhanced the surface condition of the tendons, provided additional bond, increased frictional resistance and mechanical interlocking, and strengthened bond strength and resistance ratio between tendons and concrete, offering valuable insights for improving bonded anchorage.

Jia et al. [22] enhanced the inner wall roughness by cutting grooves into the sleeve’s inner wall, thereby increasing friction between the bonding medium and the sleeve interface, reducing creep and slippage of the medium, and elevating anchoring efficiency. It was discovered that the deeper and more closely spaced the grooves were, the higher was the anchoring efficiency. Additionally, roughly treating the sleeve’s inner wall could improve the uniformity of the anchorage force on multiple tendons, enabling the tendons to be stressed synergistically and making the anchorage more stable.

In addition to the aforementioned anchorage improvement methods, some researchers have proposed new tendon end modification methods. Arnautov et al. [28] suggested a potted anchorage system for CFRP tendons by splitting the tendon ends to increase the frictional contact area and enhance the bonding capacity. May [21] put forward an anchorage scheme with end-split tendons to address scenarios where bonded anchorage length is limited. Experiments proved that the slippage of the end-loose tendon anchorage was less than that of ordinary tendons, indicating better bond strength in this scheme.

In summary, bonded anchorage transfers shear stress through the bonding effect at the tendon–adhesive interface, which inflicts less damage on the tendons and achieves superior anchoring results. Bonded anchorage can be applied to multi-tendon anchorage, with the bonding medium effectively distributing tension, enabling the tendons to work together cohesively, and offering extensive applications. Moreover, there is plenty of research on bonded type anchorage, generally focusing on changing the shape of the anchor sleeve’s inner cavity and altering relevant parameters of the anchorage system to design new types of anchorage.

### 2.3. Composite Anchorage

Concerning the bonded anchorage devices for large tonnage, multi-tendon anchor cables, there are some issues such as poor synergy among the tendons, tendency for slip in the internal tendons, and low anchorage efficiency. Given these challenges, a single anchoring method cannot reliably anchor large tonnage, multi-CFRP (carbon fiber reinforced polymer) tendon anchor cables. To address this, the concept of composite anchorage devices was developed.

**Figure 7:**
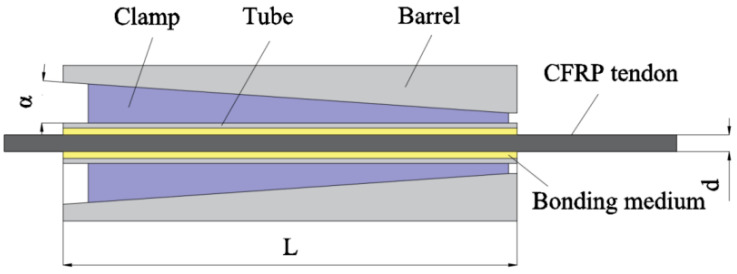
Composite anchorage.

Composite anchorage, also known as clamp bonded anchorage, combines mechanical clamped anchorage with bonded anchorage. It consists of an anchor barrel, clamp, tube, and bonding medium, as shown in Figure 7. The tendons are connected to the tube through the bonding medium in the anchorage, and the outside of the tube is clamped by the clamp. Since the clamping force does not directly act on the tendons, the risk of clamping damage is reduced. Meng et al. [29] conducted experiments that demonstrated the shared tensile force on the CFRP tendons by the steel tube and the bonding medium, which provides buffering and protection. Additionally, the combination of the three components disperses the local shear stresses on the CFRP tendons at the anchorage area, preventing shear damage caused by concentrated shear stress overload.

**Figure 8:**
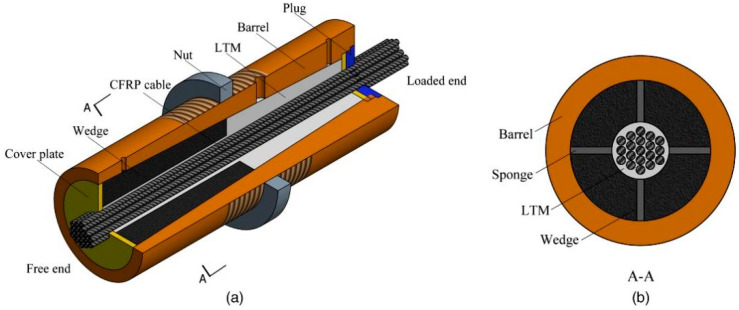
Traditional tandem type composite anchorage. ((**a**): Half section view, (**b**): Cross section).

Composite anchorage can be categorized into tandem type and parallel type, based on the different parts involved in the mechanical clamping action. In the traditional tandem type of composite anchorage clamp (also known as wedge), as shown in Figure 8, it is installed at the free end of the bonded part of the anchorage [30]. The free end bears higher radial compressive stress, which fully utilizes the anchoring effect of the free end and helps alleviate the concentration of compressive stress at the loading end, thus preventing shear damage. However, the clamping part can only exert its full clamping effect when the anchorage slips more, which may increase the risk of bond failure.

Different from the traditional anchorage, Zhuge et al. [31] designed a new type of tandem composite anchorage, where the extrusion part and the bonding part work together. The free end is clamped to each tendon end using an extruding anchor, and the tendon bundles are dispersed in the bonding medium through a wire dividing plate. Preload force is applied to the tendons, causing the extrusion anchors, wire dividing plate, and bonding medium to be tightly connected. When the tendons are subjected to force, the extrusion anchor pushes the splitter plate to press the medium tightly onto the tendons, creating a strong adhesive force at the tendon–adhesive interface, thus achieving the anchoring effect. This design enhances the performance of the bonded portion. However, it is complicated and costly to make.

**Figure 9:**
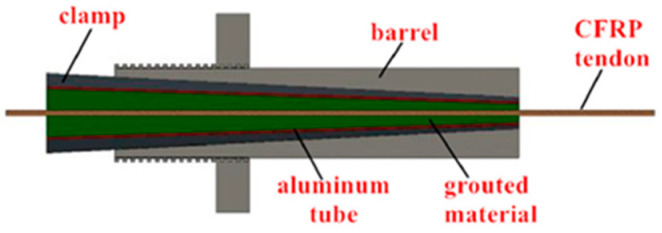
New parallel composite anchorage21.

The mechanical clamping portion of parallel composite anchorage is distributed throughout the bonding region, and it acts simultaneously with the bonding portion. Mei et al. [21] designed a new parallel composite anchorage, as shown in Figure 9. The anchor barrel of this anchorage has a slight inclination difference of 0.1° from the clamp, creating a small gap near the loaded end to reduce stress accumulation. The tendons and bonding medium are wrapped by an aluminum tube, which effectively improves the distribution length of pressure on the tendons at the clamping place due to the small hardness of the aluminum tube. As a result, the circumferential stress of the bonding medium is evenly distributed. The effects of preload force, anchorage length, taper angle, and bonding material on the anchorage performance were investigated, and the anchorage parameters optimized. Sun et al. [32] found that the preload force could improve the internal fit of this anchorage to some extent. The tilt angle difference effectively avoids stress concentration, but increasing the tilt angle difference may lead to slip generation, so the optimal tilt angle difference is set to 0.1°. A larger inclination angle of the fixture can effectively reduce circumferential stress. The increase in anchorage length can reduce anchorage slip, but to meet dimensional requirements, an anchorage length setting method of 30–40 d (where d is the diameter of CFRP tendons) is proposed. Furthermore, this anchorage design was applied to multi-tendon anchorage [33] and showed good synergy between tendons, uniform distribution of tension force, and a positive anchorage effect.

Although composite anchorage has a high anchorage efficiency coefficient, the size of the anchorage parts is typically larger, limiting its widespread use in engineering applications. It is generally only utilized in cases involving large tonnage and multi-tendon anchoring. There is relatively less research on the design of new composite anchorage systems; however, there is ample focus on studying its related parameters, indicating significant potential for further research in this area.

A comprehensive comparison of the characteristics of the three types of anchorage presented in Table 2 leads to the conclusion that the bonded anchorage exhibits a superior anchoring effect compared to the clamping anchorage, which is currently more widely used. The composite anchorage demonstrates the best anchoring performance and is suitable for the anchorage of cable-stayed bridges with large spans. However, further improvements are necessary to enhance the understanding of its mechanical properties.

### 2.4. Anchorage Damage Mechanisms

The damage mechanisms for each of the three types of anchorage are unique yet share some similarities. Regardless of the type of anchorage used, the ultimate form of damage is expected to be filamentary pull-off damage. This type of damage occurs in the non-anchored section of the tendons, where the carbon fiber filaments disperse and fracture unevenly, which is considered the most desirable form of damage. This form of damage indicates that the reinforcement maximizes the tensile strength, and the anchorage is well anchored.

Mechanically clamped anchorage can experience various forms of damage, including crushing damage, slip damage, and fiber cutting damage. Excessive inclination angle of the wedge-shaped clamp can lead to excessive extrusion pressure on the tendons, resulting in tendon crushing failure. When integrated clamps are used, the longitudinal edges of the clamps can cut into the tendons, causing splitting and crushing of the fibers. Therefore, it is crucial to design wedge-shaped clamps with appropriate inclination angles and rounded edges. Additionally, implementing protective measures on the surface of the tendons, such as aluminum or copper tubes, can help prevent crushing damage. Slip damage can occur in two forms: soft slip and dynamic slip. Soft slip failure happens when the tensile stress is low and the anchorage design is inadequate. In this case, the anchorage undergoes a gradual transition from static friction to dynamic friction, resulting in a slow pulling out of the tendons from the wedges. Dynamic slip, on the other hand, occurs when the tensile stress is excessively high, causing a rapid pulling out of the tendons from the wedges and leading to severe damage on the tendon surface. Increasing the coefficient of friction of the contact surface, adjusting the contact area, and moderately increasing the normal pressure can effectively prevent slip damage. Wedge anchoring of steel tendons can be achieved by using anchor cylinders with threaded inner surfaces; however, this method is not applicable to CFRP tendons. Unlike steel tendons, CFRP tendons cannot redistribute stresses at the sharp edges of the anchorage through yielding. As a result, stress concentration occurs, leading to fiber cutting damage [34]. When the cutting damage accumulates to a certain level, the anchorage fails. Therefore, in the design of clamped anchorage that contacts the tendons, it is important to avoid sharp edges as much as possible and use inner surface cutting indentation instead of threaded surfaces.

The damage forms of bonded anchorage can be categorized into two types: shear damage and slip damage. Shear damage typically occurs near the loading end of the anchorage and exhibits a neat fracture pattern. This type of damage is commonly caused by excessive radial extrusion and is more prevalent in non-straight anchorages. When designing and manufacturing this type of anchorage, problems such as excessive inclination, poor tendon alignment, uneven tendon distribution, and inadequate anchoring length can lead to stress concentration and shear failure of the tendons. Slip damage includes slip between the tendons and bonding medium, as well as slip between the medium and anchor sleeve [35]. It is characterized by the tendons and medium slipping out of the anchor sleeve and can occur in any bonded anchorage. Insufficient bond strength at the interfaces or insufficient stiffness of the medium can cause slip damage. Straight anchors are particularly susceptible to slip failure due to their low radial pressure, resulting in reduced adhesive force. Debonding occurs when the load reaches a certain level. Non-straight anchorages increase the radial pressure due to the presence of inclination, which improves the bonding force. However, they can still be influenced by factors such as anchorage length, media viscosity, barrel wall roughness, and media stiffness. Insufficient bonding and friction primarily contribute to slip occurrence, while media stiffness affects load-bearing deformation, leading to slip. If the medium stiffness is insufficient, the tendons drive the medium to deform in the anchor sleeve. Once the elastic deformation range of the medium is exceeded, the anchorage begins to fail, causing the medium and tendons to dislodge from the sleeve opening. Additionally, applying a certain degree of preload to the tendons helps increase bonding and reduce anchor slip.

In composite anchorage, the sleeve and bonding medium inside alleviate the radial compressive stress exerted on the tendons by the anchor cup, making slip damage the typical form of damage. For tandem composite anchorage, when the tension force is small, the bond and friction force between the bonding medium and tendons bear the tension force, with peak stress occurring at the loading end. As the tensile force gradually increases, the bonding force decreases, and the free end clamp begins to play a role. At this point, the main anchorage function is taken up by the radial pressure, friction, and mechanical grip at the free end, resulting in peak stress transferring to the free end. In parallel composite anchorage, the clamp acts as a clamping mechanism throughout the entire process. With increasing tension force, the radial force, friction force, and mechanical grip force generated by the clamping effect also increase. Slip damage in composite anchorage is mainly influenced by the surface form of the tendons, the properties of the bonding medium, and the shape of the inner casing. Threaded tendons provide improved friction and grip force at the interface between the tendons and the bonding medium compared to smooth tendons, effectively reducing slip at this interface. Bonding mediums with higher compressive strength exhibit better bonding compatibility and effectiveness [36]. Using a bonding medium with better deformation resistance can significantly reduce deformation-related slip. Straight inner sleeves are prone to slipping during the pulling process, while conical sleeves closely match the clamp, forming a self-locking mechanism that reduces slip. Improvements in these factors can effectively prevent slip damage in composite anchorage.

## 3. Novel Detection Technologies and Modeling

**Figure 10:**
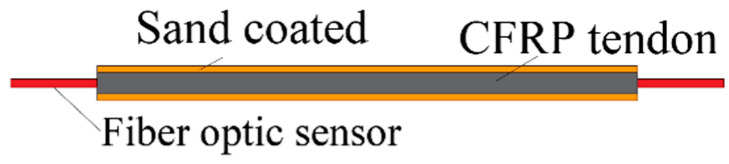
Arrangement of fiber optic sensor.

In order to study the stress distribution and damage behavior of CFRP tendon anchorage, researchers have developed new techniques for stress, strain, and damage detection, as well as established relevant mathematical models. The traditional method of measuring longitudinal stress and strain in CFRP tendons often involves locally attaching strain gauges to the surface of the tendons or sleeves. However, this method can only measure local stress and strain and does not provide a complete representation of the mechanical state of the entire tendons. Additionally, the strain gauges may become debonded or damaged during the measurement process, leading to experimental failure or errors. Züst et al. [37] utilized fiber optic sensors to measure longitudinal strains in CFRP tendons. These sensors are integrated inside the tendons along their length, as shown in Figure 10. By normalizing the strains, the stress concentration factor at each location can be estimated. Experimental results showed that the stress and strain in the free section of the CFRP tendons remained constant, while stress surges and concentration occurred in the anchorage section, which aligned with the simulation findings.

CFRP tendons have low ductility, and their fracture is the result of accumulated internal damage. The damage is transient and cannot be directly observed. Ju et al. [38] employed acoustic emission technology to collect energy and frequency data of high-energy signals during the tensile damage process of bonded anchorage tendons. They established a correlation between the damage pattern and acoustic emission results through RA-AF analysis, enabling the study of the damage mechanism of the anchorage. Three forms of damage were identified for bonded anchorage, and their corresponding RA-AF analysis results are presented in Table 3.

The high-decibel signal has the lowest percentage in the tendon stretching process but possesses the highest energy percentage. The presence of this signal indicates the occurrence of a few extremely severe damages throughout the stretching process, which serve as the primary cause of anchor failure. Thus, the damage of the bonded anchorage is sudden and unpredictable.

**Figure 11:**
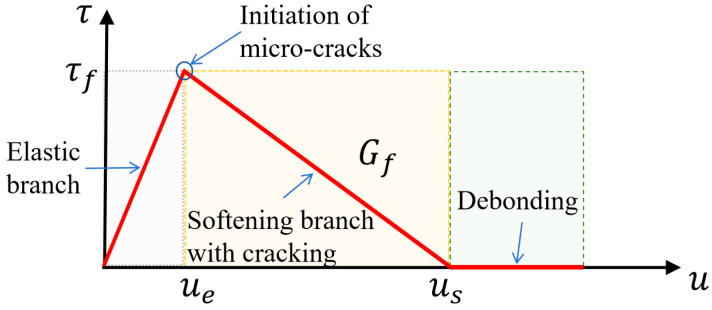
Trilinear bond slip modeling of CFRP tendons at the first anchoring interface.

Xie et al. [39] developed a generalized theoretical mathematical model for stress distribution in bonded anchors, providing stress distributions for tendons, sleeves, and bonding medium for various anchor shapes during anchoring. Additionally, Xie et al. [40] discovered a trilinear bond-slip law when analyzing the mechanical behavior of CFRP tendons by considering interfacial damage and extension at different loading stages, as depicted in Figure 11.

The three lines represent different loading stages at any position of the first interface of the anchorage, with specific values related to the nature of the bonding medium. To illustrate, consider the loading end of the anchorage. When the load is applied to the CFRP tendons, the loading end enters the first stage, which is an elastic stage. In this stage, the bond stress increases linearly with the amount of slip at the loading end. When the slip reaches $u_{e}$, the position reaches the ultimate bond strength $\tau_{f}$. At this point, micro-cracks begin to occur at the position and proceed to the next stage with increasing load. The second stage is the softening stage, during which the bonding medium begins to soften and microcracks emerge, which subsequently spread to the free end. The bond stress then decreases gradually with the increase of slip. The third stage of the process begins when the slip reaches $u_{s}$. At this point, bond failure begins to occur at this location, leaving only residual stress provided by the mechanical connection and friction between the bonding medium and CFRP tendons. It should be noted that the residual stress does not change with increasing slip. The loading end will exhibit an earlier stage of softening and bond failure compared to the free end. When bond failure occurs at the free end, the first interface is completely debonded, and the anchorage fails completely.

## 4. Impact of Non-Ideal Conditions on Anchorage Performance

Most of the current research on CFRP tendon anchors has primarily focused on static load experiments conducted under ideal conditions. However, in practical applications, anchorage is often exposed to non-ideal conditions, such as cyclic loading, impact, high temperature, moisture infiltration, and other unfavorable factors. Studying the effects of these factors is crucial for improving anchor design and prolonging service life.

CFRP tendons experience significant tensile stresses in large-span cable-stayed bridge applications. Additionally, factors like external traffic and wind can cause continuous fluctuations in axial tensile stress, leading to fatigue damage. Fatigue damage occurs due to the continuous formation and propagation of microcracks in components subjected to cyclic loading. Therefore, the design of large-span bridges and anchorage devices should consider the effects of cyclic loading. Elrefai et al. [41] conducted an experimental study on wedge anchorage systems under different cyclic loads to assess their fatigue performance. The fatigue life of wedge anchorage is primarily influenced by the stress range of cyclic loading. A larger stress range leads to a lower fatigue life, and there is a logarithmic-linear relationship between the two. When the fatigue limit of the anchorage is within 10% of its ultimate load capacity, the component can achieve an infinite fatigue life. Xie et al. [42] investigated the fatigue performance of bonded anchorage under cyclic loading and found that when the stress amplitude is within 10% of the ultimate load capacity of the anchorage system and the maximum stress is within 50% of the ultimate load capacity, cyclic loading facilitates the synergistic action of the anchorage and stabilizes its performance. However, high loading frequencies may result in a significant increase in temperature during the early and middle stages of loading. Furthermore, Xie et al. [43] analyzed the structural response of composite anchorage under cyclic loading and concluded that a high stress ratio and low stress level promote the synergistic action of composite anchorage members, thereby stabilizing damage in the anchorage zone and delaying further development. In contrast, bonded anchorage accelerates slip damage under high-stress ratio cyclic loading. Compared to bonded anchors, composite anchors exhibit better fatigue resistance.

Bridges can be subjected to impact effects caused by vehicle collisions or neighboring cable breakage during operation. Additionally, bridges in mountainous regions may face accidents such as mudslides and rockfalls. These incidents can result in sudden increases in impact load on cables and anchorage systems. Fang et al. [44] conducted a comparative experiment involving longitudinal impact and static tensile tests on bonded anchorage and found that impact actions can cause slip or even complete tendon detachment, significantly reducing the bond strength and reliability of the anchorage system. Wang et al. [45] investigated the transverse impact response of CFRP cables and their bonded anchorage systems and observed temporary slip occurring within the bond medium. After the slip, a new equilibrium is reached, and the system retains a certain load-carrying capacity.

In extreme weather conditions or special usage scenarios, anchorage systems may be exposed to rain, flooding, and other moisture-related issues, leading to rust, corrosion, and shortened service life. Even under normal conditions, atmospheric moisture can have adverse effects on anchorage systems. Therefore, it is essential to study the impact of moisture on anchorage performance. Xie et al. [46] used water immersion methods to regulate moisture in the anchorage system and analyzed its structural response under cyclic loading with wet conditions. The study found that moisture leads to hydrolysis of the chemical bond between tendons and the bond medium, resulting in the formation of microcrack damage at the interface and degradation of bond performance. The severity of the damage increases with higher water absorption in the anchorage zone. The water absorption rate shows a linear relationship with the square root of the immersion duration, highlighting the importance of drainage performance during design and construction of anchorage systems. Zhu et al. [47,48] found that water immersion accelerates stress redistribution at the bond interface of the anchorage system, with peak stress transferring from the loading end to the free end. This exacerbates bond fatigue failure.

Cable-stayed bridges are susceptible to fire incidents during both operation and construction phases. In 2014, during the construction of Chishi Bridge in Hunan Province, a fire caused by welding errors resulted in the breakage of nine cable-stayed cables. Similarly, in 2016, a truck spontaneously caught fire while passing through Sucunba Bridge in Sichuan Province, leading to damage of five cable-stayed cables. The high temperatures generated during combustion pose a significant risk to cable anchorage. As crucial load-bearing components, cables and their anchoring systems should possess sufficiently high-temperature resistance and exhibit good recovery performance after exposure to high temperatures. Researchers Su and Fang conducted studies on the mechanical properties of bonded anchorage systems at high temperatures [49] and after exposure to high temperatures [50]. The findings revealed that as the treatment temperature increased, the chemical adhesion between the tendons and the bond medium gradually decreased, resulting in an increase in slip. At this stage, the bond was primarily provided by interfacial friction and the mechanical occlusion of the embossed ribs on the surface of the tendons and the bond medium. However, the high temperature can lead to the partial decomposition of the resin matrix, causing deterioration in the shear resistance of the embossed ribs on the tendon’s surface. This deterioration results in the destruction of mechanical occlusion and further increases in slip.

To summarize, the performance of anchorage systems in cable-stayed bridges can be affected by various non-ideal conditions, including cyclic loading, impact, moisture exposure, and high temperatures. When subjected to cyclic loading, fatigue damage can occur due to the formation and propagation of microcracks. The design of large-span bridges and anchorage devices should consider the effects of cyclic loading on fatigue performance. Impact events, such as vehicle collisions or neighboring cable breakage, can cause slip or tendon detachment in the anchorage system, reducing bond strength and reliability. Moisture-related issues, such as rain and flooding, can lead to rust, corrosion, and degradation of bond performance in anchorage systems. Water absorption in the anchorage zone can accelerate stress redistribution and exacerbate bond fatigue failure. During fire incidents, high temperatures can decrease the chemical adhesion between tendons and the bond medium, leading to increased slip and reduced performance of the anchorage system. Understanding the impact of non-ideal conditions is crucial for improving anchorage design and ensuring the safety and longevity of cable-stayed bridges. Ongoing research and development are necessary to enhance the resistance and performance of anchorage systems in these challenging scenarios.

## 5. Conclusions

(1) Three types of anchoring methods are typically used with CFRP tendons: clamping anchorage, bonded anchorage, and composite anchorage. Clamping anchorage can be efficiently and conveniently applied to the anchorage of a single tendon, but in the face of a multi-tendon cable strand bearing large heavy loads, it is difficult to ensure that the tendons in the cable maintain a uniform state of force. Bonded anchorage can effectively distribute the force of multi-tendon anchoring, and the effect is good for multi-tendon anchoring, but generally requires a longer anchoring section. The new composite anchorage can combine the comprehensive advantages of clamping type and bonded anchorage, with a higher anchorage efficiency coefficient, and has potential application prospects in large tonnage, multi-tendon anchorage. However, there has been relatively little research on the design of new composite anchors, and further consideration of various working conditions is needed for more in-depth design and development.

(2) The development of a new composite anchorage should focus on making the stress distribution inside the anchorage uniform while the peak value should be small. Uniform distribution of stress in the anchorage can make all parts of the anchorage give full play to its role and improve the life of the anchorage. Excessive peak stress will exacerbate the damage of the anchorage system, which is not conducive to taking full advantage of the performance of tendons. Second, a new type of anchorage should ensure good multi-tendon coordination performance in order to prevent the internal tendons from uneven stresses producing slip failure. In addition, the design of a smaller anchorage size should be considered in terms of economy, space, and efficiency.

(3) The new composite anchorage should be analyzed and investigated by using new testing technology for stress, strain distribution, and damage mode. For example, the stress and strain distribution of the entire anchorage area tendons can be obtained by embedding a fiber optic sensor inside the tendons, exploring the anchorage performance, and reducing measurement errors. By utilizing acoustic emission technology, the energy and frequency data of high-energy signals can be collected to conduct in-depth research on the damage mechanism of the anchorage, etc.

(4) Most of the existing anchorage design research focuses on the mechanical properties of static load stretching, while the anchorage is not subjected to a single force in practical applications, and the working environment is also complex and diverse. Many adverse conditions can have adverse effects on the anchorage performance. The new composite anchorage also needs to consider dynamic research and the impact of non-ideal environmental factors such as high, low temperature, and humidity on the anchoring system.

## Figures and Tables

**Table 1:** Commonly used materials for anchors.

Alloy Steel Categories	Characteristic	Applications
40 Cr	Good economic efficiency	Suitable for medium-sized cable anchorage production
35 CrMo	Good hardenability	Suitable for anchorage components with large section sizes
42 CrMo	High strength, high hardness and good machinability	Suitable for making ultra-high strength cable anchors
40 CrNiMo	High impact properties, high hardness, poor machinability	For anchorage components with high impact requirements

**Table 2:** Characteristics and applications of various types of anchorages.

Types of Anchorages	Characteristics	Applications
Clamping anchorage	Easy to install and reusable, but more damaging to the CFRP tendons.	small amounts of CFRP tendons
Bonded anchorage	Stress distribution can be controlled by changing the sleeve structure. It is easy to produce, but it takes some time for the bonding medium to cure. The bonding medium is less damaging to the CFRP tendons, and the two are more compatible.	multi-bar anchoring; large and medium load conditions
Composite anchorage	Combining the characteristics of the above two types of anchorages gives good anchoring performance, generally larger size, complex installation and production, higher process requirements.	multi-bar anchoring; ultra-large load conditions

**Table 3:** Relationship between damage patterns and RA-AF analysis results.

Forms of Damage	Specific Manifestations	RA-AF Analysis Results
Pullout damage	Slip between tendons and medium	RA > AF
Partial debonding damage	Slippage between medium and anchor sleeve	RA ≅ AF
Tensile damage of tendons	Dispersed fiber damage of tendons	RA < AF

## Data Availability

No new data were created or analyzed in this study.
